# Relationship Between Nutrition Knowledge and Dietary Intake: An Assessment Among a Sample of Italian Adults

**DOI:** 10.3389/fnut.2021.714493

**Published:** 2021-09-13

**Authors:** Maria Luisa Scalvedi, Laura Gennaro, Anna Saba, Laura Rossi

**Affiliations:** CREA-Council for Agricultural Research and Economics, Research Centre for Food and Nutrition, Rome, Italy

**Keywords:** nutrition knowledge, eating habits, nutrition education, dietary guidelines, Italy

## Abstract

**Background:** Assessing nutrition knowledge provides useful information especially if coupled with the self-perception of nutrition knowledge that could lead to bias and personal conviction. The objective of this study was to assess nutrition knowledge (NK) and its relationship with eating habits in a group of adults.

**Methods:** A cross-sectional study with the administration of self-reported questionnaires was conducted on a sample of 591 parents (43 ± 5.82 years old) of primary school pupils recruited from the municipality of Rome (urban) and province (rural). The fieldwork was carried out in May 2017. An indicator to evaluate adherence to Italian dietary guidelines was developed. ANOVA (Welch's ANOVA in case of unequal variances) test and chi-squared test were used fixing the significance level at 5% (*p* < 0.05).

**Results:** The percentage of correct answers to nutrition knowledge was 46%, with the expert recommendation section having the highest percentage (59%). The majority of the respondents (66%) were confident that they had a high level of nutrition knowledge. In 37% of the sample, nutrition knowledge and self-perception nutrition knowledge levels were found to be associated. A total of 40% of the sample showed eating habits congruent with nutrition knowledge level. In the investigated sample, living in rural areas, being young, and having low school education level were factors associated with low nutrition literacy or/and unhealthy eating habits.

**Conclusions:** This study provided a demonstration that an assessment based on a multidimensional and multilevel approach is helpful to identify knowledge gaps and to profile critical segments to put in place targeted policy interventions.

## Introduction

Nutrition policy relied on consumer knowledge since the information of the public through education and dietary guidelines influences the behavior of people to make better choices (1). Among determinants of food choices of individuals, nutrition knowledge (NK) is considered as one of the factors affecting food intake (2). To investigate the impact of the NK effect, the General Nutrition Knowledge Questionnaire (GNKQ) was developed and validated by Parmenter and Wardle (3) and widely used in several groups of population and different settings (2, 4, 5).

Written questionnaires were frequently used to measure NK. The correctness of the responses relies on participant literacy, a relevant factor to be considered when target groups have low levels of education and socioeconomic status (6). According to Spronk et al. (7), methodologies and information collected through NK varied widely, with some assessments measuring general concepts and others focusing only on some specific nutrients such as fat or fiber. As the importance of assessing NK in nutrition education programs is recognized, it appears essential to use questionnaires that accurately evaluate the constructs intended to be measured, in particular, when the objective of the measurements is the relation between NK and diet quality (8). The individual cognitive process of nutrition information is influenced by the differences between objective knowledge (i.e., consolidated information acquired from qualified sources without personal interpretation) and subjective knowledge that occurs when people do not accurately perceive their level of competency (9).

Several studies investigated the impact of knowledge on food choice and consumption habits, showing that subjective knowledge is a stronger driver of consumer behavior than objective knowledge (10–15). As one of the factors influencing food choices, NK positively impacts the adoption of healthy eating habits (8). According to Wardle et al. (16), the highest knowledge corresponds to the highest adherence to nutritional recommendations, at least for selected foods such as fruit, vegetables, and fat. However, the growing exposure to web information and biased self-perception of NK contribute to increasing false beliefs (9). According to Alba and Hutchinson (17), consumers are overconfident, meaning that they are convinced to know more than they do.

According to Miller et al. (18), individuals will change their diets appropriately when they get accurate information about what they should eat and they should know the effects of foods consumption on health. Several studies addressed knowledge effects on dietary intake and the broad range of consumer attributes and behaviors related to foods such as attitudes, perceptions, and choices (7). The mechanism by which NK affected dietary behaviors is complex and non-univocal. Food choices and nutritional intake are determined by the awareness of individuals about food and by the self-perception of the importance of balanced meals. Nutritional awareness has a direct effect on diet quality and is related to socioeconomic factors, in particular, education and income that influence the nutritional awareness-diet quality relationship (19). It is assumed that NK will lead to an improvement of the diet by providing individuals the necessary information about choosing healthy foods, preparing and consuming these foods as recommended in dietary guidelines (20), and on the health consequences of eating unhealthy foods (5, 21). GNKQ was developed for adults (3) and the evaluation of NK was frequently carried out in different settings, such as hospitals (20) and schools (22). NK assessment in schools could be related with nutrition literacy assessments on teachers (4) and on parents to evaluate their influence on children behaviors (23, 24) also in consideration of the socioeconomic and working status of parents (25). Moreover, schools could be used simply as recruitment recipients of adults as a sample of the general population.

In Italy, NK assessment was carried out in a limited number of studies, despite the documented gap between nutritional recommendations (26, 27), food consumption pattern (28), and nutritional status of the population (29–31). As shown in a study carried out by Mazzocchi et al. (32), Italian consumers claimed for nutrition education, in particular, school education (88%), public information campaigns (81%), and nutrition information on meals provided by canteens (64%); in addition to that, 56% of the interviewed considered that the lack of knowledge on nutrition is a determinant of overweight and obesity.

In the light of this scenario, we believe that NK assessment can play an important role in understanding the nutrition information needs and awareness in population groups, hypothesizing the existence of a relationship between NK and adherence to dietary recommendations.

The purpose of this study is to measure the NK level in a sample of parents and to show the process of development and use of an innovative approach that combines the objective and subjective nutrition knowledge (S-NK) and dietetic profiles. The hypothesis is that NK has an impact on adherence to nutritional recommendations. In the intention of the authors, this approach would be able to profile the segments of the population that are more in need of interventions.

## Materials and Methods

### Setting

A cross-sectional study with the administration of self-reported questionnaires was carried out in May 2017. The setting of the study was the primary schools selected according to the following inclusion criteria: (i) absence of recent nutrition educational activities involving parents to avoid bias in knowledge evaluation; (ii) to be placed in urban and rural contexts, as defined by Eurostat classification (33). The assessment was carried out in four schools, two situated in Rome Municipality (urban area) and two in municipalities located in rural areas (Anzio and Artena). A convenience sample of 800 responses (200 per school) was planned to evaluate differences related to the degree of urbanization and considering a dropout of 30% as occurred in the study of Bonaccio et al. (34). A researcher involved in the study directly interacted with the teacher that was the reference person of the survey in the school. To reach the fixed numbers of respondents, all parents of 40 classes that fulfilled the mentioned inclusion criteria (10 per school) were involved in the survey without exclusion criteria. The teachers acted as motivators asking the children to bring home the questionnaires to be filled in by the parent usually dealing with food purchases and meals preparation. Data were collected in hard copies and then transferred in soft support using Microsoft Excel® mask. Data input was carried out by research assistants involved in the granting project (www.fruttanellescuole.gov.it). Before starting the data collection, participants were informed about the objective of the research and the consequent statistical analysis. Participation in the study was fully voluntary and anonymous and subjects could withdraw from the survey at any time and for any reason. This study was conducted according to the guidelines of the Declaration of Helsinki, and all procedures involving research study participants were approved by the participating Boards of schools. The assessment did not involve any invasive procedure nor induced any changing of dietary patterns. Therefore, the study did not require approval by the ethics committee.

### The Adaptation of NK Questionnaire

The work carried out by Bonaccio et al. (34) that validated NK Questionnaire in the Italian context was capitalized for the scope of this study. The NK Questionnaire (25 questions) evaluates three areas of knowledge: (i) recommendations of dietary expertise (NK1—four questions); (ii) calorie/nutrient composition of selected foods (NK2—18 questions); and (3) dietary risk factors for diseases (NK3—three questions). In addition to NK questions, sociodemographic information was collected along with self-reported weight and height to calculate body mass index (BMI) and define the ponderal status of respondents (35). In fact, according to Merrill et al. (36), self-reported anthropometric measurements in adults can be used for weight classification purposes. Moreover, we considered important the inclusion of this information in this assessment to detect if knowledge influenced the ponderal status, a controversial topic that deserves consideration in the light of the non-univocal relationship observed (34, 37). In April 2017, the validated Italian questionnaire was slightly revised reformulating few questions to adapt the answers to the recommendations of the Italian Food-Based Dietary Guidelines (FBDG) (19) summarized in Supplementary Material, part B. Changes in the NK Questionnaire were in section 3 (dietary risk factors for diseases). Specifically, alcoholic beverages (including wine) were added as dietary risk factors for cancer in question E1, in line with recommendations of the directive no. 8. Legumes, nuts, and fish were added as protective food items for cancer (question E1) and cardiovascular diseases (question E2) in line with directives no. 3 and no. 5. Finally, a question on the knowledge of the association between food habits and preventing diabetes was added taking into account directive no. 6.

To confirm the sensitivity of the questions and ensure the validity of the outcomes, a pretest was carried out. A panel of experts of different backgrounds (chemists, biologists, statisticians, and medical doctors) checked for the question construction, to avoid common errors and confusing wording and to validate the understandability of questions. Feedbacks from the expert panel were integrated into the final version of the questionnaire. An example of modification carried out was the denomination of some food items (e.g., in question C4 “full fat” changed in “higher fat products”). After this pilot test, the questionnaires were distributed to the schools.

The resulting questionnaire (I-NK, Supplementary Material, part A) consisted of closed-ended, multiple-choice, and yes/no questions. The scoring system used for NK measurements was a +1 point for a correct answer, 0 points for “do not know,” and −1 point for wrong answers (34).

### Integrative Modules: Adherence to Italian Guidelines Indicator, Subjective Nutritional Knowledge, and Healthy Eating Habits

The NK Questionnaire was coupled with additional modules aimed to complete the profile of behavior of respondents such as eating habits, S-NK, healthy eating, and lifestyle elements. Specific indicators were developed from these assessments. Eating habits were evaluated by adapting the food frequency questionnaire used by the Italian National Institute of Statistics (38). Specifically, the frequency of consumption of 18 food and drink groups was recorded with a categorization at a five-point scale (from “never” to “more than once per day”). Hence, an Adherence to Italian Dietary Guidelines Indicator (AIDGI) was created with a procedure similar to Benedetti et al. (39). AIDGI was based on a qualitative frequency scale and provided a synthetic evaluation of the adherence to a healthy diet as defined in the dietary guidelines. For each food group, the following scores were assigned: +2 points in case of frequency of consumption in line with recommendations, 0 points in case of frequency of consumption very far from recommendations, and +1 points in the intermediate condition, meaning not far from the recommendation, but not corresponding to it. AIDGI was calculated as the sum of 18 group scores. For example, for the groups “fresh fruit” and “vegetables,” the maximum score (2 points) was set for “more than once a day”; score 1 was assigned to option “once a day”; and 0 score was assigned to the other reported intakes.

S-NK was measured by three items based on a seven-point Likert scale (1: extremely disagree/7: extremely agree), according to Gámbaro et al. (15): (i) People I know consider me a nutrition expertise); (ii) Compared to most other people, I know many things about the nutritional properties of foods; and (iii) I know pretty well how to evaluate foods and their nutritional properties.

Questions about lifestyle and healthy eating, such as quality of breakfast (drinking only coffee or having adequate foods and nutrient intake), physical activity (sedentary habits or energy-consuming leisure time activities), water consumption (appropriate quantity of water and modality of consumption, e.g., during the meals or out of the meals), and frequency of body weight measurement were included, due to their relevance respect to the recommendations of Italian FBDG (19). Food and nutrition sources of information were also asked. These questions were designed by the authors to further evaluate the distance of knowledge of respondents from the recommendations. Data coming from these questions were processed using descriptive statistics without applying a scoring system.

### Variables Categorization and Interpretation

The distribution of objective NK score was categorized in tertiles and the S-NK average of each item was aggregated in three levels: low (1–3), medium (4), and high (5–7). Similarly, the distribution of AIDGI scores was grouped in three classes (based on the following tertiles): low—far from dietary guidelines recommendations, medium—partially met dietary guidelines recommendations, and high—adherence to dietary guidelines recommendations.

### Data Cleaning and Statistical Analysis

The overall number of questionnaires filled in was 641 out of the 800 questionnaires sent to the families, with 20% of the respondents withdrawing the participation in the study. The questionnaires were checked for the completeness of the answers, and 50 questionnaires (6%) resulted in missing data that were not consistent with validity criteria. The validity of questionnaires was defined based on the completeness of the sociodemographic section and with <10 missing answers for the NK questionnaire (out of 25 questions) since NK assessment was the key aspect of the study. After data cleaning, the final sample included 591 questionnaires.

The ANOVA was carried out to measure the influences between phenomena. The ANOVA test is particularly useful in the case where the objective is an assessment of the impact of a factor on a specific response (40). Specifically, it aims to test whether variability in a variable is attributable to one or more factors. We applied one-way ANOVA to check if a potential explanatory factor, showing different values in subgroups of the sample, is relevant in explaining differences in means (41).

The ANOVA (Welch's ANOVA in case of unequal variances) was undertaken to analyze the difference between mean scores of NK (global and specific), in subgroups of sociodemographic and ponderal status variables; the chi-squared test was used to investigate the relationships between categorical variables (NK, S-NK, and AIDGI) and sociodemographic characteristics. Cross-tabulations provided the size and profiling of specific segments. A significance level at 5% (*p* < 0.05) was fixed.

Statistical analysis was carried out using IBM SPSS Statistics for Windows, version 25. Armonk, NY: IBM Corp.

## Results

### Characteristics of the Sample

After data cleaning, the final sample included 591 valid questionnaires. The response rate of this assessment was in line with expectations. Higher family participation occurred in urban (79%) than in rural schools (69%).

Most of the respondents were females (80%). Two-thirds of the sample were <45 years old (Table 1). Younger respondents were most common in rural than in urban areas (49 vs. 24% of under 40 years old people, *p* < 0.05).

**Table 1:** Sample characteristics (*n* = 591).

	***n***	**%**
Gender		
Male	113	19.2%
Female	475	80.8%
Age group		
18–35	56	10.0%
36–40	141	25.2%
41–45	189	33.8%
46–50	135	24.2%
51 and above	38	6.8%
Education		
None	3	0.5%
Secondary school (Lev1)	81	13.8%
Secondary school (Lev2)	253	43.1%
Graduate and postgraduate	250	42.6%
Activity status		
Homemaker	96	16.3%
Unemployed	27	4,6%
Retired	1	0,2%
Worker	47	8,0%
Office worker	168	28.5%
Intellectual, scientific, and highly specialized professions	156	26.5%
Other	94	16.0%
Area		
Urban	316	53,5%
Rural	275	46,5%

A high level of education was declared by 43% of respondents, especially in urban areas (59 vs. 24% in rural areas, *p* < 0.05). Coherently, highly specialized professions resulted more common in urban than rural settings (37 vs. 15%, *p* < 0.05), while workers and housekeepers were significantly prevalent in rural contexts (respectively, 12 vs. 5% in urban, and 27 vs. 7% in urban, *p* < 0.05). The questions related to nutrition and lifestyle showed that almost half of the respondents declared to have a breakfast consistent with Italian dietary guidelines (46.4%) and to drink water in adequate quantity and modality (46.0%). The majority of the sample (77.4%) declared to perform physical activity at least once a week. Weight assessment was carried out at least once a week by 25.9% of the respondents. More than 40% of the respondents used to get nutrition information from radio/TV and magazines and books. Institutional information, either from governmental bodies or medical sectors, resulted less valued (around 20%) than family/friends exchange of information (30.6%) and newspapers or specialized websites (around 35%), 19% of the respondents referred to general websites, 17.3% indicated scientific journals, and 10.5% considered schools as a source of nutritional information.

### Nutrition Knowledge Assessment

The average NK score was 44.1, corresponding to an NK rate (percentage of correct answers) equal to 46% (Table 2). The theoretical maximum was achieved only in section NK1 Experts' recommendations (11 points). In section NK2—Food content and calories, there was found the highest gap between the maximum measured score (50) and the theoretical maximum (62). Differences were found among sections. The highest NK rate was achieved in NK1—Experts' recommendations section (59%), followed by NK2—Food composition and NK3—Diet and diseases association (both 44%).

**Table 2:** Nutrition knowledge scores.

**Descriptives**	**NK1-experts' recommendations**	**NK2-food content and calories**	**NK3-diet-disease associations**	**Total NK**
Min in the sample	−8.0	−16.0	−9.0	−15.0
Max in the sample	11.0	50.0	19.0	74.0
SD	2.8	10.6	5.1	14.6
Theoretical max (*b*)	11.0	62.0	23.0	96.0
Mean (*a*)	6.5	27.5	10.1	44.1
NK rate (*a*)/(*b*)	59.1%	44.4%	44.0%	46.0%
**Sociodemographics**	**NK1-experts' recommendations**	**NK2-food content and calories**	**NK3-diet-disease associations**	**Total NK**
	**Mean value**	**Mean value**	**Mean value**	**Mean value**
**Gender**				
Male	6.5	28.7	9.8	44.9
Female	6.5	27.2	10.2	44.0
*p*-value	0.850	0.189	0.388	0.537
**Age group**				
18–35	5.9	23.5	10.2	39.6
36–40	6.1	25.2	9.1	40.5
41–45	6.8	28.6	10.0	45.4
46 and above	6.9	30.4	11.2	48.5
*p*-value^*^	0.024	0.000	0.004	0.000
**Area**				
Urban	6.9	30.8	10.5	48.2
Rural	6.0	23.7	9.7	39.5
*p*-value^*^	0.000	0.000	0.066	0.000
**Education**				
None and secondary school (Lev1)	5.2	20.1	9.3	34.5
Secondary school (Lev2)	6.3	25.8	10.0	42.0
Degree and postgraduate degree	7.2	31.7	10.6	49.5
*p*-value^*^	0.000	0.000	0.100	0.000
**Activity status**				
Homemaker	5.6	21.3	8.8	35.7
Unemployed	5.5	22.4	9.5	37.4
Worker	6.0	21.4	9.3	36.6
Office worker	7.0	28.8	10.5	46.3
Intellectual, scientific, and highly specialized professions	7.0	33.5	10.8	51.3
Other	6.4	26.0	10.2	42.7
*p*-value^*^	0.001	0.000	0.097	0.000

* *Calculated performing the equality of means hypothesis test ANOVA test or Welch's t-test in case of unequal variances*.

#### NK Results and Relationship With Demographics

Age has been found to have an impact on NK: the elderly respondents showed significantly higher scores than the youngest in the total NK, NK2, and NK3 sections. As expected, the highest educational levels and intellectual jobs were associated with better NK scores, except for NK3. Higher NK scores were observed in urban contexts (total, NK1, and NK2) than in rural areas.

#### NK1 Results: Recommendations of Experts

**Figure 1:**
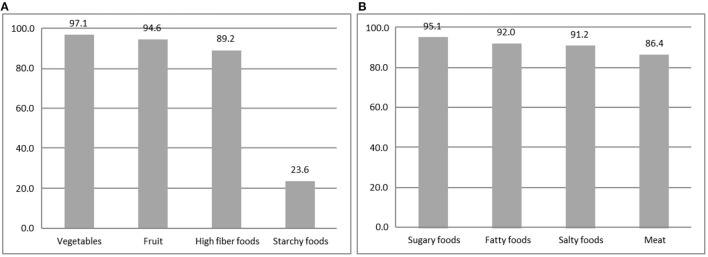
Recommendation of NK1 Expert—percentage of the correct answers of selected items. **(A)** Food to increase. **(B)** Food to limit.

Results of the NK1 section (Figure 1) showed that the recommendations to increase the consumption of fruit, vegetables, and fiber and to limit the intake of salt, sugar, and fat were well known by a large part of the respondents (almost 90%).

#### NK2 Results: Food Content and Calories

The lowest knowledge level assessed in this study was related to the starchy food recommendations. Only 23.6% of the sample provided the correct answer, while half of the respondents (48%) were convinced that the experts recommended limiting the consumption of these food products (data not shown). Another relevant evidence coming from this section not graphically showed, is related to fruit and vegetable intake. More than half of the respondents (54%) did not know the “five a day” recommendation (41), despite a large number of information initiatives in the latest years to increase fruit and vegetable consumption. The advice of limitation of saturated fatty acids consumption was known by almost six respondents out of 10. Nutrient composition section results (Table 3) showed that 63.7% recognized nuts as a source of fats and 45.2% considered nuts not included in starchy food.

**Table 3:** NK2 nutrient composition of food.

**PANEL A-food and added sugar content**	**%**	**PANEL E-food and proteins content**	**%**
Freshly squeezed orange juice-LOW	91.5	Legumes - HIGH	83.8
Canned fruit—HIGH	90.8	Fruit -LOW	78.5
Fruit Juice—HIGH	88.1	Chicken - HIGH	68.3
Ice-cream—HIGH	71.6	Cheese - HIGH	63.9
Tomato ketchup—HIGH	70.3	Butter-LOW	50.3
**PANEL B-food and fat content**	**%**	**PANEL F-food and fiber content**	**%**
Cured meat—HIGH	97.3	Breakfast cereals—HIGH	89.9
Legumes—LOW	93.7	Eggs—LOW	73.3
Pasta—LOW	93.5	Broccoli—HIGH	71.1
Cheese—HIGH	88.2	Red meat—LOW	70.3
Fried eggs—HIGH	85.7	Chicken—LOW	69.8
Margarine—HIGH	81.4	Fish—LOW	65.4
Honey—LOW	77.6	Nuts—HIGH	49.1
Bread—LOW	72.7	Banana—HIGH	43.3
Nuts—HIGH	**63.7**	Potatoes—HIGH	19.1
**PANEL C-starchy foods**	**%**	**PANEL G-food and saturated fat content**	**%**
Rice—YES	89.3	Margarine—HIGH	64.4
Pasta—YES	85.8	Whole milk—HIGH	61.4
Meat—NO	76.2	Mackerel—LOW	57.2
Cheese—NO	72.3	Nuts—LOW	46.1
Butter—NO	66.2	Red meat—HIGH	45.1
Nuts—NO	**45.2**	Olive oil—HIGH	**31.7**
**PANEL D-food and salt content**	**%**	**PANEL H-healthy alternative to red meat**	%
Canned anchovies—HIGH	93.2	Legumes—YES	92.7
Sausages—HIGH	92.7	Fish—YES	90.7
Pasta—LOW	88.5	Canned meat—NO	85.4
Frozen vegetables—LOW	78.1	Cheese—NO	**65.8**
Cheese—HIGH	77.9	Nuts—YES	**46.9**
Red meat—LOW	69.3		
Breakfast cereals—HIGH	**10.2**		

*Ranking of correct answers (low values in bold)—% value*.

It is worth noting that only 10% of respondents recognized breakfast cereals as a source of salt, although Italian Guidelines since 2003 (25) pointed out that this product is a “hidden” source of salt. Interestingly, almost all respondents recognized lentils (92.7%) and fish (90.7%) as a healthy alternative to red meat, but much fewer respondents were aware of the fact that cheese could not be considered a healthy alternative to red meat (65.8%). Results on nuts were even worsening with only 46.9% of respondents that recognize this food group as a healthy source of proteins alternative to red meat. Another false information addressed in Italian Guidelines and not known by respondents is related to brown sugar, that is, 62% of the respondents considered brown sugar healthier than white sugar.

**Figure 2:**
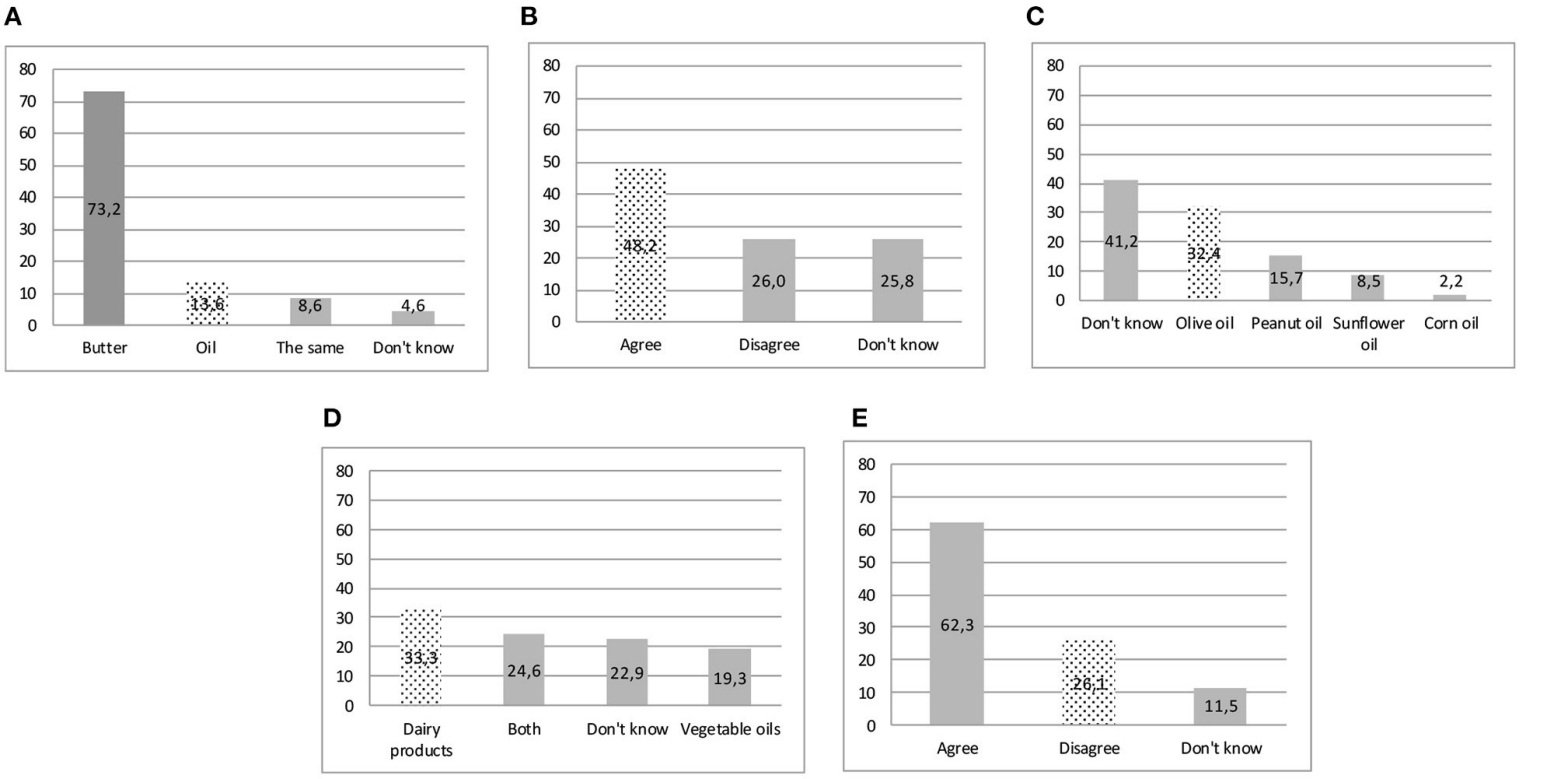
Nutrient composition of food—correct answers in the dotted bar. **(A)** Highest calorie-food. **(B)** Some foods contain a lot of fat but no cholesterol. **(C)** Oil containing mostly monounsaturated fat. **(D)** Saturated fat could be found mainly. **(E)** Brown sugar is a healthy alternative to white sugar.

Fatty food profile is not well known by respondents (Figure 2). A large majority of respondents (73.2%) claimed that butter is more caloric than oil, and 51.8% did not know (25.8%) or were uncertain (26.0%) that high-fat products could not contain cholesterol. Only 32.4% recognized olive oil as a source of monounsaturated fatty acids, and only one-third of the respondents identified dairy products as a source of saturated fatty acids.

#### NK3 Results: Diet and Diseases Association

**Figure 3:**
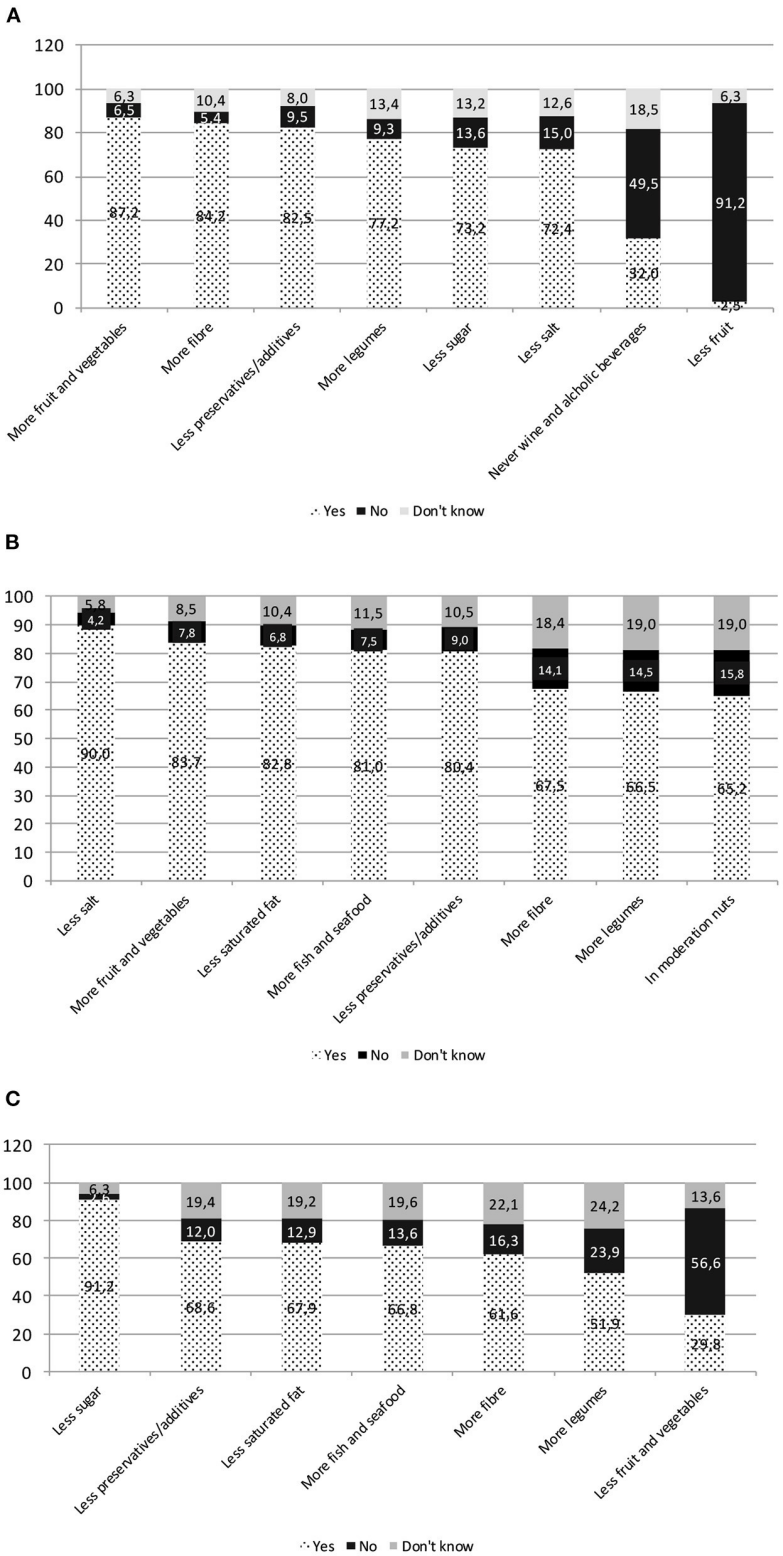
NK3 diet–disease relationship. **(A)** Food intake helping to reduce the chance of getting certain kinds of cancer. **(B)** Food intake helping to prevent heart disease. **(C)** Food intake helping to prevent diabetes.

Answers on the diet–disease relationship (Figure 3) showed that alcoholic beverages were not considered a risk factor for cancer by almost half of respondents, and 18.5% declared they did not know the correct answer. Dietetic risk factors for cardiovascular diseases resulted well known. As for diabetes, low intake of sugar was recognized as a preventive factor by almost the totality of respondents, and also saturated fats were correctly considered risk factors for diabetes by a large part of the sample (67.9%).

Other food products consumption effects on diabetes were less known: high intake of fiber and legumes were not recognized as preventive dietary habits by 38.4 and 48.1% of the sample. The limitation in the intake of fruit and vegetables by diabetics, even though it is false, is claimed as true by around one-third of respondents.

### The Nutritional Knowledge Misperception Map

The majority of the sample resulted were confident in their NK with 66.2% of the respondents considering themselves to be highly expert, 21.3% medium expert, and only 12.0% non-expert. NK and S-NK levels resulted associated (*p* < 0.05). Yet, only 36.9% of respondents perceived their NK consistently with NK score (see the gray cells along the diagonal in Table 4). On the other hand, half of the sample (51.3%) overestimated their NK, with 18.5% having high self-perception of knowledge with low NK scores. This overconfident segment included subjects mostly living in a rural area (66 vs. 44% in the total sample, *p* < 0.05), with low educational level (32.4 vs. 13.8% total sample, *p* < 0.05), and being housekeeper (31.5 vs. 16.3% *p* < 0.05) or unemployed (7.4 vs. 4.6% *p* < 0.05).

**Table 4:** Objective vs. subjective nutrition knowledge: the gap map.

**NK level**	**Subjective**			
	**Low (1–3)**	**Medium (4)**	**High (5–7)**	**Total**
Objective				
Low (<38)	**6.1%**	8.8%	18.5%	33.5%
Medium (39–51)	4.1%	**6.8%**	24.0%	34.9%
High(>51)	1.9%	5.8%	**24.0%**	31.6%
Total	12.1%	21.4%	66.5%	100.0%

*Percentage of the total sample (valid cases n = 588). Gray = coherence area*.

### The NK Adherence to Italian Dietary Guidelines Map

Food consumption frequency provided a picture of the eating habits of participants (Table 5). Frequencies exceeding the recommendations were observed for processed meats, salty snacks, sugary beverages, and alcoholic drinks including wine and beer. On the other hand, milk and yogurt, fruit and vegetables, and legumes frequencies of consumption that did not meet the recommendations, being consumed to a less extent than required according to Italian FBDG (19).

**Table 5:** The frequency of consumption of food groups.

**Food groups**	**More than once per day**	**Once per day**	**Few times per week**	**Less than once a week**	**Never**
1. Bread, pasta, rice	17.3	50.5	27.6	3.9	0.7
2. Processed and cured meat	1.4	6.3	53.8	31.1	7.4
3. Poultry	1.7	6.5	69.5	18.9	3.4
4. Red meat	0.9	4.8	50.3	37.0	6.9
5. Milk and yogurt	14.1	49.7	18.3	8.3	9.5
6. Dairy products	2.4	10.7	59.3	21.5	6.1
7. Eggs	0.9	2.4	53.8	38.7	4.3
8. Fish and fisheries products	0.8	1.9	63.3	27.7	6.3
9. Potatoes	1.9	2.2	44.2	48.3	3.4
10. Vegetables	37.0	36.2	23.2	3.2	0.3
11. Legumes	3.2	5.6	57.2	29.0	4.9
12. Fresh fruits	42.7	31.8	17.4	4.8	3.3
13. Nuts	5.0	11.6	27.1	34.8	21.6
14. Cakes and sweet snacks	4.1	13.9	35.0	39.1	7.8
15. Salty snacks	1.2	3.8	25.6	42.7	26.8
16. Sugary drinks	3.4	6.1	17.2	34.8	38.5
17. Beer and wine	0.3	8.3	20.6	27.9	42.9
18. Other alcoholic beverages	0.0	0.0	4.2	24.8	71.0

*The color of the cell indicates the Adherence to Italian Dietary Guidelines (AIDGI) scoring: white cells, score = 0; light gray cells, score = 1; and dark gray cells, score = 2*.

The ponderal status assessment showed that about half of the respondents (53.8%) had BMI values in the normal range; overweight was present in 28.9% and obesity in 10.7% of the sample. NK score was associated with ponderal status (*p* < 0.05). Obese respondents had NK scores lower than subjects with normal BMI (respectively, 37.4 vs. 45.3, *p* < 0.05). Specifically, the knowledge gap was mostly due to the content of nutrients of food (23.0 vs. 29.5, *p* < 0.05).

NK resulted strongly associated with AIDGI (*p* < 0.05). High NK level was found in the group of respondents with high AIDGI level (42 vs. 32.2% in the sample) and, symmetrically, a high concentration of individuals with low NK levels resulted in the group of low level of adherence (38.8 vs. 32.2%).

Segmenting the sample by both NK and AIDGI, consistencies were showed in 40.4% of subjects (see gray cells of Table 6) having concordant levels of indicators.

**Table 6:** The NK-Adherence to Italian Dietary Guidelines Indicator (AIDGI) map.

	**Adherence to Italian Dietary Guidelines Indicator (AIDGI) level**			
	**Low (1–3)**	**Medium (4)**	**High (5–7)**	**Total**
Objective NK level				
Low (<38)	**12.5%**	12.7%	7.0%	32.2%
Medium (39–51)	10.7%	**15.8%**	9.2%	35.7%
High(>51)	9.0%	11.1%	**12.1%**	32.2%
Total	32.2%	39.6%	28.3%	100.0%

*Percentage of the total sample (valid cases n = 513), in gray the coherence area*.

This map allows further investigations mainly to identify the sociodemographic characteristics and eating habits of critical segments. For instance, the low level of NK and AIDGI segment (12.5%, *n* = 64) includes people living in rural areas (56.1 vs. 46.5% in the total sample), having low educational level (19 vs. 13.5% in the total sample), being young (<36 years, 17.9 vs. 10%; 36–40 years 35.7 vs. 25.2%), and housekeeper (21.9 vs. 16.3%), unemployed (12.5 vs. 4.6%), and manual worker (15.6 vs. 8.0%). Looking at eating habits, low consumption of vegetables, fresh fruits, legumes, and white meat and high consumption of red meat and cheese characterize this segment (data not shown). On the other hand, the map highlights “inconsistent” segments, such as the subjects with a low level of NK and a medium or high level of AIDGI (respectively, 12.7 and 7% in the sample) or under the diagonal with a low level of AIDGI although with a medium or high level of NK (respectively, 10.7 and 9% in the sample).

## Discussion

This study is aimed to show the development and use of the NK measurement coupled with sections aimed to evaluate the dietetic profile of respondents. The results of this assessment are of interest because while the NK questionnaire was already used in Italy (34), the integration with other information—such as the adherence to the Italian dietary guidelines and S-NK—is a novelty at our best information. The exercise performed in this study accomplished the prefixed objective because we were able to profile the groups in the general population that are particularly in need of nutritional interventions. In fact, our data showed also that often people with false beliefs are convinced to be competent and that a low level of nutrition information reflects a dietary pattern, not in line with recommendations. Some of these findings were confirmed by other studies, some others provided new aspects and were open to further discussion and analysis on the complex topics of nutritional information and dietary behaviors of consumers.

As mentioned, we used a very similar NK questionnaire validated in Italy by Bonaccio et al. (34). The percentage of correct answers of present NK assessment was 46% higher than those measured by Bonaccio et al. (34) (40%, average number of correctly answered questions 37.2 of 92). The discrepancy could be explained considering several factors: the different socioeconomic contexts (central region vs. south region), the period of the survey (2017 vs. 2009), and the higher education level in our sample.

In this study, NK scores resulted strictly related to the degree of urbanization, age, education, and working status. In particular, the sample represented the structural duality between rural and urban areas, as intended in the study design. The association between NK and education and age was confirmed in other studies (34, 37, 43, 44), most of them cross-sectional, with a convenience sample of adults and in some cases including specific population groups such as athletes. The relationship between NK and education level should be taken into consideration in designing information plans. The analysis of answers to the NK questionnaire showed that in the assessed sample, recommendations of experts were better known than food composition and diet–disease relationship. Several recommendations reported in the current (19) and past Italian FBDG (45) are still not known such as brown sugar being “healthier” than white sugar. Another element of non-knowledge emerging in this study is related to the basic nutritional aspects related to fats, especially in terms of caloric contents and composition of fat-source foods. The assessment of NK showed a clear difference among consolidated long-term recommendations concerning the most recent correlations of dietetic risk factors and non-communicable diseases. In our sample, the relationships between diet and cardiovascular diseases were better known than those between nutrition and cancer. It is important to point out that alcohol was recognized as a risk factor for cancer only by one-third of respondents. This could be because the public health recommendation on alcohol consumption has changed recently (46), and the avoidance of alcohol consumption to prevent cancer was not yet known by the respondents of this study, and probably not yet widespread. The impact of NK on dietary intake received limited research attention despite the relevance of this investigation (7). Specifically, most studies, cross-sectional and with a convenience sample, reported a significant and positive association between NK and some aspects of dietary intake, with few studies reporting negative associations, and approximately one-third failed to observe any association (7, 37).

We confirmed an association between dietetic profile and NK scores. In our sample, a significant proportion of individuals with nutritional habits highly adherent to recommendations had also a high NK score; the relation is biunivocal with subjects having unhealthy nutrition habits showing low NK scores. Looking at the diagonal of the NK-AIDGI map, a proportion of 40% of respondents were classified as having high NK and high adherence to recommendations. As to the segments classified outside the diagonal, the reasons for the “inconsistency” could be related to the complexity of food choice determinants that include physiological components, cultural and social pressures, and personality characteristics (47). In the same way, high adherence to recommendations associated with low NK could be related to consolidated healthy eating habits derived from cultural and familiar heritage, even in the presence of scarce nutrition literacy. Further research needs to be carried out to better understand the causal relationship between food choice determinants and NK.

However, consistently with the aims of this study, both the maps showed critical segments revealing vulnerable groups. The overconfident segment (low objective NK and high S-NK, 19% of the sample) and the segment including subjects with a low level of knowledge and eating habits (12%) presented common socioeconomic characteristics: young age, living in rural areas, unemployed, and economically inactive, with low education level. Interestingly, the urban context showed higher NK scores and education and lower objective/S-NK divide concerning the rural context. These findings reflect the fact that rural areas would require stronger and focused efforts in terms of educational intervention. If these data will be confirmed in a larger study, education interventions should take into account these societal aspects.

In this sample, people that know more about nutrition tend to eat better and consequently to have normal weight, as demonstrated by the relationship between NK and ponderal status. A concept that seems obvious but not so linear and not always confirmed in other studies. A review performed by Barbosa and coworkers (44) found that high scores of NK may influence the adoption of healthy food habits, but not always do these scores show an association with body weight. In the same way, Kliemann et al. (37) could not find a relationship between NK and ponderal status in a study carried out in the United Kingdom. Instead, the other mentioned Italian study (34) proved that the highest scores of NK are associated with adherence to Mediterranean dietary patterns and a lower prevalence of obesity.

In the light of mentioned novelties, this study has limitations. The opportunistic sampling would not permit a generalization and extrapolations at a broad level should be made cautiously. Another limitation is related to the dietary assessment based on a food frequency questionnaire that does not provide an accurate food consumption measurement. However, the objective of this study was not a consumption evaluation to measure calories and nutrients; the inclusion of a food frequency section in NK assessment was aimed to describe food habits, providing a qualitative assessment of consumption patterns aimed to measure the adherence to dietary recommendations. For the first time and with a pioneering approach, we developed the AIDGI, an indicator based on a qualitative frequency scale that needs to be improved, even if it seems reasonably appropriate for the scope of the protocol.

The innovative aspect and strength of this study are the two directions of the assessment allowing to build a multidimensional framework (from knowledge to behavior) as well as a multilevel framework from micro (i.e., knowledge about the role of specific food groups in nutrition or frequency of consumption of an individual food group) to macro information (global assessment of both NK and adherence to dietary guidelines). As such, it appears a helpful potential assessment approach for educational campaigns either as a benchmark as well as an evaluating instrument.

This work contributed to the debate on the role of NK as a key element able to stimulate behavior changes toward healthier eating habits. NK as part of the most general health literacy represents an asset to be considered along with S-NK and eating habits to identify specific groups of the population. The results encourage future research. Performing a national study to refine the instrument developed in this explorative study as a support for planning educational interventions could be of high interest in the public health nutrition context. With this approach, nutrition education activities and programs would have the highest impact, reaching people that are more in need than usually are those less motivated to change. General consideration on the provision of recommendations and the source of nutrition information could be provided. This study showed that institutional bodies are not considered as the reference sources by the individuals involved in the survey. If confirmed by a larger analysis, it would represent a key aspect to be faced, also considering that specialized web communication resulted frequently consulted by respondents. The issue of web communication and, in general, the communication that uses means different from classical instruments, such as guidelines, leaflets, newspapers, or TV, needs to be addressed by educators and communicators either in nutrition or in other science sectors.

## Data Availability Statement

The raw data supporting the conclusions of this article will be made available by the authors, without undue reservation.

## Ethics Statement

Ethical review and approval was not required for the study on human participants in accordance with the local legislation and institutional requirements. Written informed consent for participation was not required for this study in accordance with the national legislation and the institutional requirements.

## Author Contributions

MS and AS: conceptualization. MS: data curation. MS, LG, and LR: investigation and methodology. MS and LR: writing—original draft. MS, LG, AS, and LR: writing—review and editing. All authors contributed to the article and approved the submitted version.

## Funding

This work was funded by the Italian Ministry of Agricultural, Food and Forestry Policies (MIPAAF), Grant D.M.97381-31/12/2016 in the framework of Italian Accompanying measures to the UE Fruit school scheme. The publication of this paper was possible thanks to the funds of the project FAOWASTE: Food waste in Italy international policies and measurements (No.23278-27.12.2019) granted by the Italian Ministry of Ecological Transition. The Funders had no role in the design, analysis, or writing of this article.

## Conflict of Interest

The authors declare that the research was conducted in the absence of any commercial or financial relationships that could be construed as a potential conflict of interest.

## Publisher's Note

All claims expressed in this article are solely those of the authors and do not necessarily represent those of their affiliated organizations, or those of the publisher, the editors and the reviewers. Any product that may be evaluated in this article, or claim that may be made by its manufacturer, is not guaranteed or endorsed by the publisher.
