# Supplementary material for: Relationship Between Nutrition Knowledge and Dietary Intake: An Assessment Among a Sample of Italian Adults
Source: Front Nutr. 2021 Sep 13;8:714493. doi: 10.3389/fnut.2021.714493 (PMC8473625; doi:10.3389/fnut.2021.714493)
Supplement: Supplementary file 1 [file Data_Sheet_1.docx]

Supplementary Material

**A. The I-NK questionnaire: Italian Nutrition Knowledge questionnaire, subjective Nutrition Knowledge, food habits**

List of the questionnaire items measuring i) nutrition knowledge related to experts ‘recommendations for a healthy diet, calories/nutrients composition of selected food products, and associations between diet and diseases; ii) subjective knowledge; iii) eating habits

| ***Experts’recommendations for a healthy diet*** |  |
| --- | --- |
|  | **C1.** What do you think the experts recommend that people should be eating for the following foods? (*eating more; eating less; no recommendation; I don’t know*)  *vegetables; sugary foods ; meat; cereals and derivates (bread, pasta, rice, …); fatty foods; high fibre foods; fruit; salty foods*  **C2.** How many servings of fruit and vegetables a day do you think experts are advising people to eat? (*1; 2, 5 or more; more than 10; I don’t know*)  **C3.** Which fats do experts recommend reducing (*monounsaturated; polyunsaturated; saturated; I don’t know*)  **C4.** Which version of dairy foods do experts advice people to eat (*higher fat products; lower fat products; saturated; I don’t know*) |
| ***Calories/nutrients composition of selected food products*** |  |
|  | **D1.** Do you think these food products are high or low in added sugar? (*high; low; I don’t know*)  *fruit juice; ice cream; fresh squeezed orange juice; tomato ketchup; canned fruit in natural juice*  **D2.** Do you think these food products are high or low in fat? (*high; low; I don’t know*)  *pasta (without sauce); legumes; fried eggs; cured meat; honey; nuts; bread; cheese; margarine*  **D3.** Do you think these food products are part of the ‘starchy foods” category? (*yes; no; I don’t know*)  *cheese; pasta; butter; nuts; rice; meat*  **D4.** Do you think these food products are high or low in salt? (*high; low; I don’t know*)  *sausages; pasta; canned anchovies; red meat; breakfast cereals; cheese; frozen vegetables*  **D5.** Do you think these food products are high or low in proteins? (*high; low; I don’t know*)  *chicken; cheese; fruit; legumes; butter*  **D6.** Do you think these food products are high or low in fibre? (*high; low; I don’t know*)  *breakfast cereals; bananas; eggs; red meat; broccoli; nuts; fish; potatoes; chicken*  **D7.** Do you think these food products are high or low in saturated fat? (*high; low; I don’t know*)  *mackerel; whole milk; olive oil; red meat; margarine; nuts*  **D8.** Do you think these food products are a healthy alternative to red meat? (*yes; no; I don’t know*)  *fish; legumes; nuts; cheese; canned meat*  **D9.** Which of these food products mainly contain saturated fats? (*vegetable oils; dairy products; both; I do not know*)  **D10.** Which of these breads contain most vitamins and minerals? (*white; wholegrain; potato flour bread; I do not know*)  **D11.** Which one do you think is higher in calories? (*butter; oil; the same; I do not know*)  **D12.** In your opinion, the oil with more monounsaturated fats is...? (*corn oil; sunflower oil; olive oil; peanut oil; I do not know*)  **D13.** In your opinion, which one of the following has the most calories for the same weight? (*sugar; bread; fruit; fats; I do not know*)  **D14. In your opinion,** polyunsaturated fats are mainly found in? (*vegetable oils; dairy products; both; I do not know*)  **D15.** Some food products are rich in fat but not in cholesterol. (*agree; disagree; I do not know*)  **D16.** A glass of fruit juice can replace a fruit. (*agree; disagree; I do not know*)  **D17.** Brown sugar is a healthy alternative to white sugar. (*agree; disagree; I do not know*)  **D18.** Margarine contains less fat than butter does. (*agree; disagree; I do not know*) |
| ***Associations between diet and diseases*** |  |
|  | **E1.** Do you think these eating habits help to reduce the chances of getting certain kinds of cancer? (*yes; no; I don’t know*)  *eating more fibre; eating less sugar; eating less fruit; eating less salt; eating more fruit and vegetables; eating less preservatives/additives; never drink wine and alcoholic beverages; eat more legumes*  **E2.** Do you think these eating habits help prevent hearth diseases? (*yes; no; I don’t know*)  *eating more fibre; eating less saturated fat; eating less salt; eating more fruit and vegetables; eating less preservatives/additives; eating more legumes; eating more fish and fishery products; eating nuts in a moderately way*  **E3.** Do you think these eating habits help prevent diabetes diseases? (*yes; no; I don’t know*)  *eating more fibre; eating less saturated fat; eating less fruit; eating less sugar; eating less preservatives/additives; eating more fruit and vegetables; eating less preservatives/additives; eating more legumes; eating more fish and fishery products* |
| ***Subjective Nutrition knowledge*** |  |
|  | **A.5** People I know consider me a nutrition expert (1: extremely disagree / 7: extremely agree)  **B5.** Compared to most other people, I know many things about the nutritional properties of foods (1: extremely disagree / 7: extremely agree)  **F3.** I know pretty well how to evaluate foods and their nutritional properties (1: extremely disagree / 7: extremely agree) |
| ***Eating habits*** |  |
|  | **B6.** How often do you habitually consume the following foods and drinks? (*more than once per day; once per day; few times per week; less than once a week; never*) |

**B. Italian Food-based dietary guidelines: summary of recommendations**

The Italian Dietary Guidelines for Healthy Eating (official name) were published in December 2019 (CREA Centro di ricerca per gli alimenti e la nutrizione (2018) Linee Guida Per Una Sana Alimentazione Italiana. Revisione 2016. Dossier Scientifico. https://www.crea.gov.it/en/web/alimenti-e-nutrizione/-/linee-guida-per-una-sana-alimentazione-2018 (accessed May 2021). The intended audience of the Guidelines is the general public. Recommendations cover all age groups from infants to the elderly, including physiological conditions such as pregnancy and lactation. There is also a focus on special requirements for people who practice sports, as well as on providing recommendations for people at increased risk of obesity and most common non-communicable chronic diseases (Cardiovascular diseases, cancers, diabetes). The Guidelines are also intended to be used by health professionals who deal with nutrition, the private sector and journalists (generalist and scientific). The main messages are intended to be the basis for school nutrition education programs. The 13 directives have been divided into four blocks. The first is related to balance (1). The second is dedicated to foods for which consumption needs to be increased, such as fruits and vegetables (2-4). The third concerns critical foods in the current diet and for which consumption should be reduced, such as fat, salt, sugar and alcohol (5-8). And the last block is dedicated to "How to" ensure a varied, safe, healthy and sustainable diet (9-13).

The 13 directives and the main recommendations included in each directive are:

| **Conceptual block** | **Directive** | **Main recommendations** |
| --- | --- | --- |
| Balance | 1. Keep your weight under control and always be active | - In case of overweigh reduce intake and increase physical activity. - Avoid very restrictive diets that exclude entire food groups. - Be careful to extreme food behaviour that could be symptoms of eating disorders. |
| Increase consumption | 2. Eat more fruits and vegetables | - Increase fruits and vegetable consumption limiting the adding of added fats and salt. - Choose seasonal fruit and vegetables varying colours. - Fruits juice cannot replace a portion of fresh fruit. |
|  | 3. Eat whole grain and legumes | - Increase consumption of fibre choosing whole grain products. - Increase legume intake as alternative to animal source foods. - Remember the importance of fibre as protective factor for non-communicable disease |
|  | 4. Drink abundant water every day | - Water must be the preferred fluid for rehydration. - Drinks at least 8 glasses of water a day, more is better. - Increase water intake during physical activity. |
| Reduce consumption | 5. Fats: select which ones and limit the quantity | - Reduce intake of saturated fat choosing foods containing unsaturated fatty acid for cardiovascular prevention. - Remember that all fats have the same caloric content. - Remember that in Italy trans-fatty acids are any more present in industrial products. |
|  | 6. Sugar, sweets and sugar sweetened beverages: less is better | - Reduce intake of sugar in favour of starchy foods - High intake of sweetened beverages is a risk factors for non-communicable diseases, including diabetes and obesity. - Remember that brown sugar, honey and fructose are not healthy alternative to sugar. |
|  | 7. Salt: less is better (but iodised) | - Reduce intake of salt and chose iodine fortified products. - Remember that several industrial products are hidden source of salt (e.g. breakfast cereals). - Remember that salt intake is an important risk factors for non-communicable disease in particular heart disorders. |
|  | 8. Alcoholic beverages: the least possible | - Avoidance of alcohol of any source including wine and beer, is the best for health. - If you decide to drink alcohol it is for your pleasure not for health; limits the quantities: not more than 1 alcoholic unit (e.g. a glass of wine) per day for female and elderly and 2 alcoholic units per day for males. - No alcohol of any type to children, adolescents, pregnant and lactating women. |
| How to do | 9. Enjoy a variety of food choices | - Remember that choosing variety of foods is a way to guarantee nutritional adequacy. - Variety does not means more foods, portions and frequencies must be adequate to energy consumption at different ages and physiological status. - Mediterranean Diet is the dietary pattern that inspire the Italian FBGDs. |
|  | 10. Follow special recommendations for target groups | - Remember that children have special needs in particular during infancy: select foods of high quality in adequate quantity. - Pregnancy and breastfeeding are physiological periods that require attention: best to think before and arrive to these moments in good health and putting in place all preventive actions needed (e.g. folic acid supplementation). - Elderly need to eat a little less because the metabolism slows down but the quality of the food must be higher without forget to maintain an active lifestyle. |
|  | 11. Be careful of dieting and misuse of dietary supplements | - Dieting is a therapeutic acts that require trained professionals; consumers should avoid to refer to non-qualified people. - Loose weigh require time and constancy, everything and immediately is not compatible with dieting. - Dietary supplements could be important in case of deficiency but never substitute a healthy diet. |
|  | 12. Food safety depends also on you | - At home, be careful to good storage of food in the refrigerator. - At the supermarket, in the grocery cart and bags separate fruit and vegetables from meat, poultry and fish to avoid cross-contamination. - Prepare the kitchen, clean the sink before and after washing and preparing fresh fruit and vegetables, use different cutting board and preparation area for meat/poultry/fish and fresh fruits and vegetables. Wash especially well between preparation of meat/poultry/fish and preparation of foods that will be eaten without cooking. |
|  | 13. Select a sustainable diet | - Avoid processed meat and reduce red meat consumption in favour of poultry or vegetal source of protein. Select fish from sustainable stocks, e.g. small fish from Mediterranean see (anchovies, sardines, mackerel, etc.); not demonise aquaculture. - Increase consumption of plant foods avoiding selection of products that require large use of external inputs for growing (e.g. high fertilizing, artificial light and heating or overseas products). - Planning, preparing, and storing food can help consumers waste less food, save money, and eat healthier food. Re-use old ingredients or leftovers in new dishes. |
